# Accuracy of echocardiography in diagnosing total anomalous pulmonary venous return

**DOI:** 10.12669/pjms.345.15766

**Published:** 2018

**Authors:** Fatima Ali, Sonia Qureshi, Muneer Amanullah, Mehnaz Atiq

**Affiliations:** 1 Dr. Fatima Ali, FCPS (Pediatrics). Department of Pediatrics and Child Health, The Aga Khan University Hospital, Karachi, Pakistan; 2 Dr. Sonia Qureshi, FCPS (Pediatrics). Department of Pediatrics and Child Health, The Aga Khan University Hospital, Karachi, Pakistan; 3 Dr. Muneer Amanullah, FRCS (General Surgery RCS Edinburgh). Department of Surgery, The Aga Khan University Hospital, Karachi, Pakistan; 4 Dr. Mehnaz Atiq, FCPS (Pediatrics), FCPS (Pediatric Cardiology). Department of Pediatrics and Child Health, The Aga Khan University Hospital, Karachi, Pakistan

**Keywords:** Total anomalous pulmonary venous return, Echocardiography, Computed tomographic angiography, Heterotaxy

## Abstract

**Objective:**

Total anomalous pulmonary venous return is an uncommon cyanotic congenital heart defect. Echocardiography is the initial diagnostic tool. Complimentary non-invasive modalities like cardiac computerized tomographic angiography and cardiac magnetic resonance imaging have replaced the need for cardiac catheterization in difficult cases. This study aimed to determine the accuracy of echocardiography in diagnosing total anomalous pulmonary venous return, and to determine the factors that may decrease its sensitivity.

**Methods:**

This was a cross-sectional study conducted at the Aga Khan University Hospital Karachi, Pakistan from January 2010 to August 2016. All patients who were diagnosed with Total anomalous pulmonary venous return on echocardiography and had subsequent confirmation either on cardiac CT angiography or surgery were included. The diagnostic accuracy of echocardiography was expressed as sensitivity. Previously described taxonomy was used to define diagnostic error. Univariate and multivariate analysis were done by logistic regression OR (95% CI) were reported to identify factors causing the diagnostic error.

**Results:**

High diagnostic sensitivity (81%) was found in isolated total anomalous pulmonary venous return and low (27%) in heterotaxy and mixed (20%) varieties. Poor acoustic windows and right isomerism were found to be significant factors responsible for the diagnostic error on multivariate analysis.

**Conclusion:**

Echocardiography can diagnose isolated total anomalous pulmonary venous return with high accuracy. Use of additional modalities may be required for a complete diagnosis in cases with mixed variety, heterotaxy and poor acoustic windows.

## INTRODUCTION

Total anomalous pulmonary venous return (TAPVR) is a rare congenital cardiac defect (CHD) which occurs as a result of defective development or early atresia of the common pulmonary vein. The incidence of TAPVR ranges from 0.6 to 1.2 per 10,000 live births, and is the fifth most common cause of CHD.^1^,^2^ TAPVR is categorized as supracardiac, cardiac, infracardiac, or mixed forms.^3^ Surgical outcomes depend upon the anatomical type of TAPVR and presence or absence of obstruction to the drainage of these anomalous veins. Therefore, defining the connection of all pulmonary veins, drainage of the confluence, and course of the vertical vein and presence of obstruction are important components of a complete diagnosis.^4^,^5^

Echocardiography is an excellent diagnostic modality, but challenges may be seen in cases with mixed or infracardiac variety, identification of obstruction and in TAPVR associated with heterotaxy syndrome or right atrial isomerism.^6^-^8^ In neonates with respiratory distress, especially those on high-frequency oscillation mode of ventilation, poor acoustic windows, spatial resolution and the subjective interpretation of the operator are additional factors limiting accurate echocardiographic diagnosis. Complimentary modalities like cardiac magnetic resonance imaging (cardiac MRI), cardiac computerized tomographic angiography (cardiac CT angiography) or cardiac catheterization may provide complete information in difficult cases.^9^-^11^ Although previous studies have emphasized the diagnostic value of echocardiography,^7^,^8^ no recent data is available.^12^

The objective of this study was to evaluate the accuracy of echocardiography in the diagnosis of TAPVR (isolated or associated with other structural heart defects) when compared to the diagnosis made by cardiac CT angiography or during surgery and to determine diagnostic errors responsible for any differences observed.

## METHODS

This is a retrospective cross-sectional study conducted at Aga Khan University Hospital, Karachi, Pakistan. Data was obtained from January 2010 to August 2016 from the medical records of patients diagnosed with TAPVR on echocardiography and had subsequent confirmation either on cardiac CT angiography or surgery. Cases with incomplete data, echocardiography without a final cardiologist’s interpretation, lack of confirmatory test (cardiac CT angiography or surgical), and cardiac CT angiography performed outside AKUH were excluded. The institutional ethical review committee granted approval for this study.

Demographic data reviewed through patient’s medical records included age, weight and gender. Echocardiography was performed on i-E33 Phillips (Andover, MA, USA), Vivid GE-9 and HD-11 Phillips machines (Fairfield, CT, USA) with 12 F transducer probe for neonates, 5 F for infants and 3.5 F for older children. Conscious sedation with chloral hydrate 40mg/kg was used when required. All projections were critically reviewed by a pediatric cardiologist. Parasternal long and short axis, apical 4-chamber, subcostal, and suprasternal views of all four pulmonary veins and their drainage were evaluated for the confluence of veins, course and flow in the vertical vein, the size of the atrial septal defect, presence of obstruction at any level and other associated anomalies. Reports and films of cardiac CT angiography were reviewed with consultant radiologist. Surgical notes were reviewed with a pediatric cardiac surgeon for specific operative findings.

The taxonomy employed by Benavidez et al^13^,^14^ to define diagnostic errors was applied.

***A) Accuracy of diagnosis by echocardiography***:

***1) Complete echocardiographic diagnosis:*** All pulmonary veins and their connection, the drainage of the confluence, and course of the vertical vein were clearly identified on echocardiography.

***2) Incomplete echocardiographic diagnosis:*** Any missing information from the above description and/or discrepancy between the echocardiographic description and diagnosis made on cardiac CT angiography or direct observation during surgery.

***B) Diagnostic error definition***:

***1)*** A diagnostic error was defined as a diagnosis that did not meet the above described definition.

***C) Diagnostic error categorization***:

***1) False-negative:*** An error that states that a finding is normal when an abnormality is present, or when a reader failed to include a significant diagnostic possibility.

***2) False-positive:*** An error that reports an abnormality when no abnormality is present or the reader overemphasized the significance of a finding.

***3) Discrepant diagnosis:*** The actual diagnosis is different from one made.

***D) Severity of error categorization: (categorized based on surgical impact)***:

***1) Minor:*** A diagnostic error or discrepancy that did not change patient management or clinical course.

***2) Moderate:*** A diagnostic error or discrepancy had an impact on management or clinical course but no impact on outcome.

***3) Major:*** Diagnostic error discrepancy had an impact on management that resulted in an adverse event.

***4) Catastrophic:*** A diagnostic error or discrepancy that contributed to death.

***E) Categorization of preventability***:

***1) Not preventable:*** The diagnostic error was not preventable because images, imaging modalities, or imaging conditions did not permit accurate diagnosis.

***2) Possibly preventable:*** The diagnostic error could have been prevented if the true diagnosis was expected while using the imaging modality and/or imaging conditions, but may also have required a different technique, modality or condition.

***3) Preventable:*** The diagnostic error was preventable if the accurate diagnosis was expected, given the available images, imaging modality, and/or imaging conditions.

***F) Categorization of the cause of the diagnostic error***:

***1) Demographic:*** This includes age, gender, and weight.

***2) Disease-related:*** This includes right atrial isomerism, associated CHD, type of TAPVR (supra, infra, cardiac and mixed varieties).

***3) Technical:*** This includes poor acoustic windows and type of machine used.

### Statistical analysis

Results are expressed in frequencies and percentages. The echocardiographic accuracy was reported as sensitivity. We could not comment on specificity as only diagnosed cases of TAPVR were included in this study. Factors associated with diagnostic accuracy were assessed by univariate analysis. Variables with a p-value <0.25 were then considered for inclusion in a multivariate logistic regression model. Subsequently, a p-value of < 0.05 was considered as significant. Adjusted odd ratios (AORs) with 95% confidence intervals (CI) were also calculated.

## RESULTS

A total of 47 patients were enrolled in this study, of which 33 (70%) were male. Ages ranged from one day to 168 months with a median age of three months. Weight ranged from 1.8 kg to 39 kg with a median of 3.5kg. Echocardiography was compared with surgical findings in 24 patients and with cardiac CT angiography in 30 patients. In seven patients in whom cardiac CT angiography was performed prior to surgery, the findings were consistent with those described in the surgical notes. 32 (68%) were with isolated TAPVR (Table-I). 15 patients had associated congenital heart defects, all of whom (100%) had a ventricular septal defect. 9 (60%) patients had double outlet right ventricle, 11 (73%) had complete atrioventricular septal defect, five (33%) had pulmonary atresia, and six patients (40%) had persistent left superior vena cava. Right atrial isomerism was seen in 12 (80%). Echocardiography missed identification of two associated congenital heart anomalies: one case of persistent left superior vena cava and one case of pulmonary atresia, while other findings were in agreement with CT angiography or surgical diagnosis. Echocardiography was found to be highly sensitive in diagnosing isolated TAPVR (81%) while in mixed variety and TAPVR associated with other CHD, the diagnostic value was low (27%) (Table-II).

**Table-I:** Types of TAPVR.

Types of TAPVR	Total TAPVR n=47 (100%)	Isolated TAPVR n=32 (68%)	TAPVR with CHD n=15 (32%)
Supracardiac	18(39)	10(31)	8(53)
Cardiac	16(33)	13(41)	3(20)
Infracardiac	8(17)	6(19)	2(13)
Mixed	5(11)	3(9)	2(13)

TAPVR: total anomalous pulmonary venous return; CHD: congenital heart defect.

**Table-II:** Echocardiographic diagnosis (frequency) and accuracy (sensitivity).

TAPVR ECHO Diagnosis	All cases TAPVR n (%)	Isolated TAPVR n (%)	Associated CHD with TAPVR n (%)	Supracardiac TAPVR n (%)	Cardiac TAPVR n (%)	Infracardiac TAPVR n (%)	Mixed TAPVR n (%)
Complete diagnosis	30(64)	26(81)	4(27)	13(72)	12(75)	4(50)	1(20)
Incomplete diagnosis	17(36)	6(19)	11(73)	5(28)	4(25)	4(50)	4(80)
Sensitivity	64%	81%	27%	72%	75%	50%	20%

TAPVR: total anomalous pulmonary venous return; CHD: congenital heart defect.

### Diagnostic error categorization

15 cases were identified as diagnostic errors. Among them 60% had discrepant diagnosis, 27% were false negatives, and 13% were false positives. Diagnostic error severity: All errors were in the minor severity category. There were no errors in the moderate, major or catastrophic severity categories. Diagnostic error preventability: 60% were not preventable, 32% were possibly preventable while 8% were preventable.

### Contributing factors to diagnostic errors

Diagnostic error was most common with the mixed variety of TAPVR (seen in 80%) with three (75%) cases having only one pulmonary vein draining separately while the other three pulmonary veins drained within the confluence. In the supra-cardiac variety, one (6%) had right sided vertical vein which was missed. In TAPVR associated with CHD, 12 (80%) had right atrial isomerism, and 75% of these had an error in diagnosis. Factors contributing to diagnostic errors were patient-related including right atrial isomerism (in 53%) and poor acoustic windows (in 47%).

With univariate analysis, factors found to be associated with diagnostic errors included male gender, portable echocardiographic machine, poor acoustic windows, right isomerism and a mixed variety of TAPVR (p-value <0.05) (Table-III). With multiple regression analysis, right isomerism and poor acoustic windows were the only factors significantly affecting diagnostic accuracy. (Table-IV)

**Table-III:** Risk factors for diagnostic errors (univariate analysis).

Variables	Odd ratio	95% CI	p-value
Age (months)	1.001	0.9858 – 1.018	0.828
Weight	1.044	0.9264 – 1.1784	0.474
Gender	0.370	0.0867 – 1.5799	0.18
Machine	0.213	0.0484 -0 .9361	0.041
Windows	0.038	0.0042 - 0.3533	0.004
Right isomerism	0.0987	0.0214 -0 .4544	0.003
Other CHD	0.2586	0.0216 – 3.0884	0.285
Supracardiac	1.835	0.5160 – 6.5274	0.348
Cardiac	2.166	0.5686 – 8.2554	0.261
Infracardiac	2.000	0.4298 – 9.3060	0.377
Mixed	0.112	0.0113 – 1.1032	0.061

CHD: congenital heart defect

**Table-IV:** Risk factors for diagnostic errors (Multivariate analysis).

Variables	Odd ratio	95% CI	p-value
Machine	0.239	0.0450 – 1.2715	0.093
Right isomerism	0.121	0.0197 – 1.2715	0.023
Acoustic Windows	0.0571	0.0051 - 0.6352	0.021

## DISCUSSION

Until the advent of non-invasive imaging modalities like cardiac CT angiography or cardiac MRI, cardiac catheterization was the adjunct to echocardiography for accurate preoperative diagnosis of anomalous pulmonary veins. The sensitivity of echocardiography in diagnosing TAPVR has been reported to be around 80%.^11^ Lee et al concluded that cardiac catheterization and angiocardiography were unnecessary, and echocardiography could accurately determine the pulmonary venous drainage site. Their study was limited by a small sample size, and they did not include mixed variety and TAPVR with heterotaxy syndromes.^6^ Huhta et al. included a mixed type of TAPVR and heterotaxy syndromes in their report and found that both situations compromise accurate echocardiographic diagnosis.^7^ They reported 97% sensitivity and 99% specificity of echocardiography in diagnosing TAPVR with atrial situs solitus, and only 71% sensitivity in TAPVR with right atrial isomerism. Other studies have also shown a diagnostic sensitivity of 87% with echocardiography.^4^,^8^ Hussain et al. demonstrated 91.6% sensitivity and 100% specificity of echocardiography in diagnosing TAPVR, but the inclusion criteria were not defined.^12^ The conclusion of most of these studies highlights the limited diagnostic accuracy of echocardiography in the mixed variety and in association with right atrial isomerism, as was demonstrated in this study. However, none of the studies report the cause of misdiagnosis nor have they analyzed error gravity and preventability.

Although the present study did not differ from what has previously been reported, the application of taxonomy has helped in a detailed evaluation with improvement in quality strategies. We have shown a high sensitivity of echocardiography in diagnosing isolated TAPVR but low accuracy in diagnosing the mixed type and in those with heterotaxy syndromes. In the mixed variety, diagnostic errors in 3 (75%) cases could have been prevented if the one pulmonary vein draining separately was visualized and reported. One neonate on a high-frequency oscillatory mode of ventilation had poor acoustic windows. Additionally, in one case artifacts made the false positive interpretation of mixed variety, which could have been prevented by checking with Doppler image or repeated studies. Among patients with heterotaxy syndromes, echocardiography missed left sided SVC in one case due to an inadequate acquisition of images.

Univariate analysis showed portable echocardiography machines, poor acoustic windows, and right atrial isomerism to be significant factors hindering complete diagnosis (p <0.05). Poor acoustic windows and right atrial isomerism were found to be significant factors in multivariate analysis as well. Proper image acquisition using echocardiography of the four pulmonary veins, their draining sites and confluence, and repeated examination in difficult patients would help in improving diagnosis. In cases with poor acoustic windows, mixed variety of TAPVR or heterotaxy syndrome, the use of additional imaging should be considered to provide complete preoperative data.

Although cardiac MRI is an excellent diagnostic modality, it is skill intensive, needs familiarity with post processing software and requires general anesthesia especially in neonates and small children.^9^ Cardiac CT angiography is relatively less time consuming, less technically demanding and can be performed under conscious sedation alleviating the need for the anesthetic team. With the newer generation multi-detector computed tomographic scanners, because of their improved gantry rotation time, table pitch and increased volume coverage, the duration of scan has been significantly reduced, and a high-resolution image of the heart is captured in less than one-third of a second. All of this has led to a marked reduction in radiation dose (0.8 mSv for a cardiac CT angiography compared to 6-10 mSv for conventional angiograms). This makes cardiac CT angiography a cost-effective and safe modality that can be used to supplement echocardiography in improving diagnostic precision.^15^-^17^

## CONCLUSION

Echocardiography is highly sensitive in diagnosing isolated TAPVR. Factors reducing its diagnostic accuracy were the mixed variety, right atrial isomerism and poor acoustic windows. Use of additional imaging modality is recommended in such cases to provide a complete diagnosis.

### Strength and Limitations of the Study

The strength includes use of taxonomy and multivariate analysis for risk factors. However the limitations of the study are small sample size, inclusion of all age groups and diagnostic precision by echocardiography decreases in older age groups because of poor acoustic windows.

### Authors’ Contribution

**FA:** Conceived, designed, data collection and manuscript writing.

**FA & SQ:** Did data collection statistical analysis and manuscript writing.

**MAm & Mat:** Did analysis, review, manuscript editing and final approval of manuscript.
